# Thanking our 2014 peer reviewers

**DOI:** 10.1186/s12943-015-0308-2

**Published:** 2015-03-31

**Authors:** Christophe Nicot, Alan Storey

**Affiliations:** Kansas University Medical Center, United States of America, Kansas, USA; ᅟ, Oxford, UK

## Abstract

The editors of *Molecular Cancer* would like to thank all the reviewers who contributed to the journal in Volume 13 (2014) by participating in the review process.

**Electronic supplementary material:**

The online version of this article (doi:10.1186/s12943-015-0308-2) contains supplementary material, which is available to authorized users.

**Cory Abate Shen**

United States of America

**Tatjana Adamovic**

Sweden

**Bharat Aggarwal**

United States of America

**Tommy Alain**

Canada

**Arthur Alberts**

United States of America

**Stefano Alcaro**

Italy

**Ramon Alemany**

Spain

**Alain Algazi**

United States of America

**Lindsey Allan**

United Kingdom

**Heike Allgayer**

Germany

**Soner Altiok**

United States of America

**Noona Ambartsumian**

Denmark

**Stefan Ambs**

United States of America

**Hesham Amin**

United States of America

**Nicola Amodio**

Italy

**Olga Aprelikova**

United States of America

**Ulrich Arnold**

Germany

**Asaithamby Aroumougame**

United States of America

**Nicola Arrighi**

Italy

**Michael Ausserlechner**

Austria

**Farrukh Awan**

United States of America

**Duncan Ayers**

Malta

**Hideo Baba**

Japan

**Ivan Babic**

United States of America

**Manuela Baccarini**

Austria

**Serena Rubina Baglio**

Italy

**Rameshwar Bamezai**

India

**Dario Barbone**

United States of America

**Betsy Barnes**

United States of America

**Mark Barok**

Finland

**Martin Barr**

Ireland

**John Barrett**

Canada

**Monica Bartucci**

United States of America

**Katia Basso**

United States of America

**Robert Bast**

United States of America

**V. Lokesh Battula**

United States of America

**James Beattie**

United Kingdom

**Philippe Becuwe**

France

**Martin Belinsky**

United States of America

**Brendan Bell**

Canada

**Marcia Bellon**

United States of America

**Mariaserena Benassi**

Italy

**Doris Benbrook**

United States of America

**Rosaria Benedetti**

Italy

**Robert Bennett**

United States of America

**Carmen Berasain**

Spain

**Michael Berens**

United States of America

**Michael Bergmann**

Austria

**Joseph Bertino**

United States of America

**Giorgio Berton**

Italy

**Edouard Bertrand**

France

**Robert Besch**

Germany

**Darell Bigner**

United States of America

**Eric Blair**

United Kingdom

**Joanne Emily Bluff**

United Kingdom

**Richard Blumberg**

United States of America

**Italia Bongarzone**

Italy

**Maria Carla Bosco**

Italy

**Hicham Bouhlal**

France

**Edward Bradley**

Canada

**Claudio Brancolini**

Italy

**Randall Brand**

United States of America

**Walburgis Brenner**

Germany

**Pnina Brodt**

Canada

**Laki Buluwela**

United Kingdom

**Fred Bunz**

United States of America

**Franco Buonaguro**

Italy

**Emanuele Buratti**

Italy

**Andrean Burnett**

United States of America

**Matthew Burow**

United States of America

**Ezra Burstein**

United States of America

**Rita Busuttil**

Australia

**Qingchun Cai**

United States of America

**Matthew Caley**

United Kingdom

**George Calin**

United States of America

**Daniele Calistri**

Italy

**Paola Campomenosi**

Italy

**Carme Camps**

United Kingdom

**Marco Candeias**

Japan

**Ida Candiloro**

Australia

**Anna Rita Cantelmo**

Belgium

**Qi Cao**

United States of America

**Bo Cao**

United States of America

**Guangwen Cao**

China

**Michele Caraglia**

Italy

**Matteo Carlino**

Australia

**Peter Carlsson**

Sweden

**Maria Carmo-Fonseca**

Portugal

**Amancio Carnero**

Spain

**Jason Carroll**

United Kingdom

**Jon Carthy**

Sweden

**Lynne Cassimeris**

United States of America

**Lina Cekaite**

Norway

**Bo Cen**

United States of America

**Guralp Ceyhan**

Germany

**Florence Chainiaux**

Belgium

**Chiranjib Chakraborty**

India

**Michael Chan**

Taiwan

**Stephen Chan**

United States of America

**Dhyan Chandra**

United States of America

**Joya Chandra**

United States of America

**Chawnshang Chang**

United States of America

**Jang-Yang Chang**

Taiwan

**Wen-Chang Chang**

Taiwan

**Pithi Chanvorachote**

Thailand

**Sally Chappell**

United Kingdom

**Srikumar Chellappan**

United States of America

**Xiaoji Chen**

United States of America

**Xin Chen**

United States of America

**Limin Chen**

Canada

**Rong Chen**

United States of America

**Yan Chen**

China

**Yunyun Chen**

United States of America

**Ann-Lii Cheng**

Taiwan

**Hua Cheng**

United States of America

**Liang Cheng**

United States of America

**Sok Ching Cheong**

Malaysia

**LUIS Chiva**

Spain

**William Cho**

Hong Kong

**Khoi Chu**

United States of America

**Wen-Ming Chu**

United States of America

**James Church**

United States of America

**Chih-Pin Chuu**

Taiwan

**Bob Clarke**

United States of America

**Anne-Marie Cleton-Jansen**

Netherlands

**Nicole Cloonan**

Australia

**David Cobrinik**

United States of America

**Yossi Cohen**

Israel

**Patricia Cohen**

United Kingdom

**Andrew Colebatch**

Australia

**Sabine Colnot**

France

**Paolo Comoglio**

Italy

**Graham Cook**

United Kingdom

**John Copeland**

Canada

**John Copland**

United States of America

**Pelayo Correa**

United States of America

**Simona Corso**

Italy

**Jack Cowland**

Denmark

**Judy Crabtree**

United States of America

**Carlo Croce**

United States of America

**Tim Crook**

United Kingdom

**Zoran Culig**

Austria

**Martine Culty**

Canada

**Artur Czekierdowski**

Poland

**Min Dai**

United States of America

**Yan Dai**

United States of America

**Ichikawa Daisuke**

Japan

**Sorab Dalal**

India

**Hien Dang**

United States of America

**Fanny Daniel**

France

**Michael Danquah**

United States of America

**Piyali Dasgupta**

United States of America

**Kaustubh Datta**

United States of America

**Ben Davidson**

Norway

**Kurtis Davies**

United States of America

**Michael Davies**

United States of America

**Pieter De Bleser**

Belgium

**Vittorio de Franciscis**

Italy

**Steven de Jong**

Netherlands

**Ivana de la Serna**

United States of America

**Rainer de Martin**

Austria

**Giuseppina De Petro**

Italy

**Anita De Rossi**

Italy

**Joris de Wit**

Belgium

**Giannino Del sal**

Italy

**Dajun Deng**

China

**Xingming Deng**

United States of America

**Chadrick Denlinger**

United States of America

**Paul Dent**

United States of America

**Punita Dhawan**

United States of America

**Lucia Di Marcotullio**

Italy

**Analisa DiFeo**

United States of America

**Anna Dimberg**

Sweden

**Han-Fei Ding**

United States of America

**Amanda Dixon-McIver**

New Zealand

**Mustafa Djamgoz**

United Kingdom

**Yoh Dobashi**

Japan

**Xiaoqun Dong**

United States of America

**Yujuan Dong**

Hong Kong

**Zigang Dong**

United States of America

**Valérian dormoy**

United States of America

**Harry Drabkin**

United States of America

**Liqin Du**

United States of America

**Wolfgang Dubiel**

Germany

**Gene Dubowchik**

United States of America

**Laurence Dubrez**

France

**Maria Duca**

France

**Thomas Duchaine**

Canada

**Stefan Duensing**

Germany

**Marlène Dufresne**

France

**Karen Dunphy**

United States of America

**Joelle Dupont**

France

**Lee Eckhardt**

United States of America

**Richard Eckner**

United States of America

**Rossella Elisei**

Italy

**Lynne Elmore**

United States of America

**Georg Enders**

Germany

**Nuray Erin**

Turkey

**Stefan Erkeland**

Netherlands

**Stephen Ethier**

United States of America

**Muller Fabbri**

United States of America

**Akos Fabian**

Hungary

**Isabel Fabregat**

Spain

**Germana Falcone**

Italy

**Xiaolong Fan**

China

**Joachim Fandrey**

Germany

**Lin Fang**

China

**Jia Fang**

United States of America

**Marianne Farnebo**

Sweden

**Maree Faux**

Australia

**Eric Fearon**

United States of America

**Felix Feng**

United States of America

**Martin Fernandez-Zapico**

United States of America

**Silvano Ferrini**

Italy

**Remond J.A. Fijneman**

Netherlands

**Cristina Fillat**

Spain

**Paul Fisher**

United States of America

**Jude Fitzgibbon**

United Kingdom

**Erik Flemington**

United States of America

**Abd AlRahman Foda**

Egypt

**Michael Föller**

Germany

**C Fordyce**

United States of America

**Patricia Forgez**

France

**Al Forrest**

Japan

**Archa Fox**

Australia

**Marc Fransen**

Belgium

**Michael Freeman**

United States of America

**Leonardo Freire de Lima**

Brazil

**Steven Frisch**

United States of America

**Buyin Fu**

United States of America

**Junya Fujimoto**

United States of America

**Kiyoko Fukami**

Japan

**Galina Gabriely**

United States of America

**Silvia Galardi**

Italy

**Andrea Galli**

Italy

**Ian Gallicano**

United States of America

**Hui Gan**

Australia

**Suthakar Ganapathy**

United States of America

**Allen Gao**

United States of America

**Yuzhen Gao**

China

**Giovanni Gaudino**

United States of America

**Maria Gazouli**

Greece

**Dirk Geerts**

Netherlands

**Sarah Gerlo**

Belgium

**Christian Gespach**

France

**David Gewirtz**

United States of America

**Debashis Ghosh**

United States of America

**Goutam Ghosh Choudhury**

United States of America

**Sean Gill**

Canada

**Doron Ginsberg**

Israel

**Antonio Giordano**

United States of America

**Jean-Antoine Girault**

France

**Munirathinam Gnanasekar**

United States of America

**Katalin Goda**

Hungary

**Hira Goel**

United States of America

**Wolfgang H. Goldmann**

Germany

**David Goltzman**

Canada

**Vita Golubovskaya**

United States of America

**Selma de Andrade Gomes**

Brazil

**Christopher Gondi**

United States of America

**Lorenza Gonzalez-Mariscal**

Mexico

**Susana Gonzalo**

United States of America

**Vidya Gopalakrishnan**

United States of America

**Margarete Goppelt-Struebe**

Germany

**Michael Gottesman**

United States of America

**Susumu Goyama**

United States of America

**Dinny Graham**

Australia

**Sheila Graham**

United Kingdom

**Sveva Grande**

Italy

**Michael Graner**

United States of America

**Tim Greten**

United States of America

**Pat Griffin**

United States of America

**Thomas Griffith**

United States of America

**Lyn Griffiths**

Australia

**Kenneth Grossmann**

United States of America

**Arnold Grünweller**

Germany

**Jian Gu**

United States of America

**Rafael Guerrero-Preston**

United States of America

**Ghiabe Guibinga**

United States of America

**Preethi Gunaratne**

United States of America

**Baoliang Guo**

China

**Mingzhou Guo**

China

**Vineet Gupta**

United States of America

**Katerina Gurova**

United States of America

**Nikolas Haass**

Australia

**Trevor Hambley**

Australia

**Paul Hamel**

Canada

**Ian Noel Hampson**

United Kingdom

**Haiyong Han**

United States of America

**Yuchen han**

China

**David Harder**

United States of America

**Johan Hartman**

Sweden

**Julia Hauer**

Germany

**Allison Haugrud**

United States of America

**Petr Hausner**

United States of America

**Matthew Hayden**

United States of America

**Nicholas Hayward**

Australia

**Nicholas Kim Hayward**

Australia

**Lori Hazlehurst**

United States of America

**Yulong He**

China

**Christopher Heaphy**

United States of America

**Joan Heath**

Australia

**Olaf Heidenreich**

United Kingdom

**Karl Erik Hellstrom**

United States of America

**Ingegerd Hellstrom**

United States of America

**Martin Hemler**

United States of America

**Kyoko Hida**

Japan

**Martin Higgs**

United Kingdom

**Masahiro Higuchi**

United States of America

**Tetsuro Hirose**

Japan

**Mary Hitt**

Canada

**Karin Hochrainer**

United States of America

**Rob C. Hoeben**

Netherlands

**Andrea Hoelbl-Kovacic**

Austria

**Alexander Hoellein**

Germany

**David Hong**

United States of America

**Liu Hong**

China

**Wanjin Hong**

Singapore

**Peter Hordijk**

Netherlands

**Weihong Hou**

United States of America

**Gillian Howell**

United States of America

**Sarah Howie**

United Kingdom

**Guochang Huang**

United States of America

**Tony Huang**

United States of America

**Robert Huebert**

United States of America

**Kam Hui**

Singapore

**Joseph Humtsoe**

United States of America

**Keun Hur**

Korea South

**Douglas Hurst**

United States of America

**Antoni Hurtado**

Norway

**R. Katherine Hyde**

United States of America

**Inmaculada Ibañez de Caceres**

Spain

**Zoya Ignatova**

Germany

**Takashi Ikejima**

China

**Evgeny Imyanitov**

Russian Federation

**Hiroyuki Inuzuka**

United States of America

**John Isaacs**

United States of America

**Sumiya Ishigami**

Japan

**Alexander Ishov**

United States of America

**Maria Izquierdo-Pulido**

Spain

**Meena Jaggi**

United States of America

**Maneesh Jain**

United States of America

**Mark Jameson**

United States of America

**Miriam Jasiulionis**

Brazil

**Junfang Ji**

United States of America

**Lijun Jia**

China

**Bing-Hua Jiang**

United States of America

**Chenchen Jiang**

Australia

**Feng Jiang**

United States of America

**Xuejun Jiang**

United States of America

**Pengfei Jiang**

United States of America

**Xi Jiang**

United States of America

**Xiaohua JIANG**

Hong Kong

**Lei Jin**

Australia

**Takashi Joh**

Japan

**Jeremy Johnson**

United States of America

**Randy Johnson**

United States of America

**Frank Jones**

United States of America

**Edgar Jost**

Germany

**Anna Margaretha Joubert**

South Africa

**Jingfang Ju**

United States of America

**Ian Judson**

United Kingdom

**Kerstin Junker**

Germany

**Humam Kadara**

United States of America

**Christoph Kahlert**

Germany

**Sham Kakar**

United States of America

**Enikoe Kallay**

Austria

**Galatea Kallergi**

Greece

**Dhan Kalvakolanu**

United States of America

**Ashish Kamat**

United States of America

**Bozena Kaminska**

Poland

**Joseph Kaminski**

United States of America

**Chunsheng Kang**

China

**Chunsheng Kang**

China

**Chi-Dug Kang**

Korea South

**Ningling Kang**

United States of America

**Jessica Kao**

United States of America

**Adam Karpf**

United States of America

**Santosh Katiyar**

United States of America

**Masaru Katoh**

Japan

**Deepak Kaul**

India

**Sukhwinder Kaur**

United States of America

**Richard Kefford**

Australia

**Kornelius Kerl**

Germany

**Santosh Kesari**

United States of America

**Ahmad Khalil**

United States of America

**Hark Kim**

Korea South

**Hyung Sik Kim**

Korea South

**Injune Kim**

Korea South

**Joon Kim**

Korea South

**Kevin Kim**

United States of America

**Kyu-Tae KIM**

Australia

**Jason Kindrachuk**

United States of America

**Csongor Kiss**

Hungary

**Uwe Knippschild**

Germany

**Fumitaka Koga**

Japan

**Gou Young Koh**

Korea South

**Eiji Kohmura**

Japan

**Mikhail Kolonin**

United States of America

**Masayuki Komada**

Japan

**Takashi Kondo**

Japan

**John Koomen**

United States of America

**Tomasz Kordula**

United States of America

**Greg Korpanty**

Canada

**Nina Korzeniewski**

Germany

**Claudia Kowolik**

United States of America

**Markus Kretz**

Germany

**Adam Krieg**

United States of America

**Natalia Krupenko**

United States of America

**Georg Krupitza**

Austria

**Lucia Kucerova**

Slovakia

**Santosh Kumar**

United States of America

**Sanjay Kumar**

United States of America

**Gopal Kundu**

India

**Siavash Kurdistani**

United States of America

**Masahiko Kuroda**

Japan

**Bernhard Kuster**

Germany

**Vijay Kumar Kutala**

India

**Yoshikazu Kuwahara**

Japan

**Young-Guen Kwon**

Korea South

**Natasha Kyprianou**

United States of America

**Raymond Lai**

Canada

**Zhi-Chun Lai**

United States of America

**Sonia Lain**

Sweden

**Imayavaramban Lakshmanan**

United States of America

**Rajkumar Lakshmanaswamy**

United States of America

**Ola Larsson**

United States of America

**Pierre Laurent-Puig**

France

**Gad Lavie**

Israel

**Brian Law**

United States of America

**Sean Lawler**

United States of America

**Yuri Lazebnik**

United States of America

**Huei Lee**

Taiwan

**You Mie Lee**

Korea South

**Po-Huang Lee**

Taiwan

**Kin wah Lee**

Hong Kong

**Susan Lees-Miller**

Canada

**NL Lehman**

United States of America

**Ulrich Lehmann**

Germany

**Dominique Leprince**

France

**Vera Levina**

United States of America

**Carmit Levy**

Israel

**Jian Jian Li**

United States of America

**Xiang-Ping Li**

China

**Long-Cheng Li**

United States of America

**Xiaoling Li**

United States of America

**Yueming Li**

United States of America

**Youjun Li**

China

**Yi Li**

United States of America

**Min Li**

United States of America

**Yang Li**

China

**Jianming Li**

China

**Zonghai Li**

China

**Evi Lianidou**

Greece

**Joshua Liao**

United States of America

**Jong-Seok Lim**

Korea South

**Chunru Lin**

United States of America

**Chin-Yo Lin**

United States of America

**Dechen Lin**

United States of America

**Ding-Yen Lin**

Taiwan

**Zongming Lin**

China

**Peter Linsley**

United States of America

**Stanley Lipkowitz**

United States of America

**Laurie Littlepage**

United States of America

**Min Liu**

China

**Bolin Liu**

United States of America

**Jinsong Liu**

United States of America

**Ling-Zhi Liu**

United States of America

**Bo Liu**

China

**Gongping Liu**

China

**Zhi-Hua Liu**

China

**Ying Liu**

China

**Yangfan Liu**

United States of America

**Zheng-Gang Liu**

United States of America

**Zhi-Ren Liu**

United States of America

**Hui-Wen Lo**

United States of America

**Kwok Wai Lo**

Hong Kong

**Joseph Loftus**

United States of America

**Bal Lokeshwar**

United States of America

**Anna Lokshin**

United States of America

**Marc Lopez**

France

**Haya Lorberboum-Galski**

Israel

**Fotios Loupakis**

Italy

**Jose Lozano**

Spain

**Hua Lu**

United States of America

**Xiongbin Lu**

United States of America

**Julian Lum**

Canada

**Amanda Lund**

United States of America

**Ji Luo**

United States of America

**Zhijun Luo**

United States of America

**Traci Lyons**

United States of America

**Lukas Mach**

Austria

**Katerina Machova Polakova**

Czech Republic

**Marcello Maggiolini**

Italy

**Kiran Mahajan**

United States of America

**Pamela Maher**

United States of America

**Renaud Mahieux**

France

**Kien Mai**

Canada

**Mala Maini**

United Kingdom

**Paraskevi Mallini**

United Kingdom

**Guidalberto Manfioletti**

Italy

**Koren Mann**

Canada

**Sandrine Marchetti**

France

**Durvanei Maria**

Brazil

**John Mariadason**

Australia

**Maria Marino**

Italy

**John Martens**

Netherlands

**Pia M Martensen**

Denmark

**Atsushi Masamune**

Japan

**Robert Mason**

United States of America

**Suresh Mathivanan**

Australia

**Yasunobu Matsuda**

Jordan

**Isao Matsuura**

Taiwan

**Michelle Matter**

United States of America

**Robert Matts**

United States of America

**Massimiliano Mazzone**

Belgium

**Dennis McCance**

United Kingdom

**John McDonald**

United States of America

**Kerrie McDonald**

Australia

**Declan McKenna**

United Kingdom

**Iain McNeish**

United Kingdom

**Barbara Melendez**

Spain

**Zhiqiang Meng**

China

**Laura Mercatali**

Italy

**Jean-Louis MERLIN**

France

**Markus Metzler**

Germany

**Antimo Migliaccio**

Italy

**Matthew Miller**

United States of America

**Ian Mills**

Norway

**Tom Milne**

United Kingdom

**Toshinari Minamoto**

Japan

**Nikica Mise**

Germany

**Akira Miyajima**

Japan

**Suzanne Miyamoto**

United States of America

**Yin-Yuan Mo**

United States of America

**Beverly Mock**

United States of America

**Faustino Mollinedo**

Spain

**Hugo Monteiro**

Brazil

**Rodolfo Montironi**

Italy

**Richard Moore**

United States of America

**Hari Narayana Moorthy**

Portugal

**Mark Morgan**

United Kingdom

**Pat Morin**

United States of America

**Sharon Celeste Morley**

United States of America

**Laura Moro**

Italy

**David Morris**

Australia

**Franck Mortreux**

France

**Justin Mott**

United States of America

**Mirna Mourtada-Maarabouni**

United Kingdom

**Caroline Moyret-Lalle**

France

**Lina Mu**

United States of America

**Naofumi Mukaida**

Japan

**Hasan Mukhtar**

United States of America

**James Mullin**

United States of America

**Teresita Munoz-Antonia**

United States of America

**Jordi Muntané**

Spain

**Satoshi Murata**

Japan

**Satu Mustjoki**

Finland

**Joe Mymryk**

Canada

**Karthikeyan Mythreye**

United States of America

**Ernest Nadal**

United States of America

**Alo Nag**

India

**Sonia Najjar**

United States of America

**Hiroyasu Nakano**

Japan

**Ichiro Nakano**

United States of America

**Harikrishna Nakshatri**

United States of America

**Kalyan Nannuru**

United States of America

**Christian Nanoff**

Austria

**Honami Naora**

United States of America

**Aziza Nassar**

United States of America

**Sathish kumar Natarajan**

United States of America

**Dirk M. Nettelbeck**

Germany

**Jochen Neuhaus**

Germany

**Daniel Neureiter**

Austria

**Thu Nguyen**

United States of America

**Bing Ni**

China

**Christophe Nicot**

United States of America

**Douglas Noonan**

Italy

**Susan Nozell**

United States of America

**Greg Oakley**

United States of America

**Eva Obermayr**

Austria

**Josiah Ochieng**

United States of America

**Takahiro Ochiya**

Japan

**Yoshinao Oda**

Japan

**Karin Oechsle**

Germany

**Yukio Okano**

Japan

**Barry O'Keefe**

United States of America

**Denise O'Keefe**

United States of America

**Soriani Olivier**

France

**Ghislain Opdenakker**

Belgium

**Véronique Orian-Rousseau**

Germany

**Francisco José Ortega**

Spain

**Majid Osman**

Sweden

**Lindsey Oudijk**

Netherlands

**Jaya Padmanabhan**

United States of America

**Jihye Paik**

United States of America

**José Palacios**

Spain

**Tanapat Palaga**

Thailand

**Moorthy Palanimuthu Ponnusamy**

United States of America

**Swati Palit Deb**

United States of America

**Joanna Pancewicz-Wojtkiewicz**

Poland

**Atanasio Pandiella**

Spain

**Carlos Panizo**

Spain

**Benedict Panizza**

Australia

**Renate Panzer-Grümayer**

Austria

**Mayra Paolillo**

Italy

**Anthony Papenfuss**

Australia

**Luis A Pardo**

Germany

**Changwon Park**

United States of America

**Seung Woo Park**

Korea South

**Won Sang Park**

Korea South

**Marina Pasca di Magliano**

United States of America

**Manish Patankar**

United States of America

**Paola Patrignani**

Italy

**James Patton**

United States of America

**Kajsa Paulsson**

Sweden

**Richard Pearson**

Australia

**Peter Pelka**

Canada

**Christopher Penfold**

United Kingdom

**Guang Peng**

United States of America

**Guangyong Peng**

United States of America

**Ignacio Perez de Castro**

Spain

**Carlos Perez-Stable**

United States of America

**Guy Perkins**

United States of America

**Rosario Perona**

Spain

**Alexander Pertsemlidis**

United States of America

**Francesca Peruzzi**

United States of America

**Ulrich Pfeffer**

Italy

**Francesca Pica**

Italy

**Julia Pickl**

Germany

**Smitha Pillai**

United States of America

**Pascal Pineau**

France

**Paolo Pinton**

Italy

**Ruben Plentz**

Germany

**Elke Pogge von Strandmann**

Germany

**Laila Poisson**

United States of America

**Gretchen Poortinga**

Australia

**Angel Porgador**

Israel

**Bellur Prabhakar**

United States of America

**Vikram Prasad**

United States of America

**Muriel Priault**

France

**Esther Priel**

Israel

**Michele Pritchard**

United States of America

**Lisa Privette Vinnedge**

United States of America

**Darwin Prockop**

United States of America

**Ron Prywes**

United States of America

**Armin Pycha**

Italy

**Chao-Nan Qian**

China

**Wenbin Qian**

China

**Nancy Raab-Traub**

United States of America

**Satyanarayana Rachagani**

United States of America

**Prakash Radhakrishnan**

United States of America

**Cláudia Rainho**

Brazil

**Krishnaraj Rajalingam**

Germany

**Darek Rakus**

Poland

**Ranju Ralhan**

Canada

**Sundaram Ramakrishnan**

United States of America

**Ramani Ramchandran**

United States of America

**Pranela Rameshwar**

United States of America

**Joe Ramos**

United States of America

**Ajay Rana**

United States of America

**Basabi Rana**

United States of America

**Katya Ravid**

United States of America

**Pradip Raychaudhuri**

United States of America

**Chiara Recchi**

United Kingdom

**Félix Recillas-Targa**

Mexico

**Roger Reddel**

Australia

**Glen Reid**

Australia

**Milan Reinis**

Czech Republic

**Jun Ren**

United States of America

**Peter Revill**

Australia

**C Patrick Reynolds**

United States of America

**Vincent Ribrag**

France

**Carmela Ricciardelli**

Australia

**Ann Richmond**

United States of America

**Piotr Rieske**

Poland

**Markus Riester**

United States of America

**Natalia Andrea Riobo**

United States of America

**Arun Rishi**

United States of America

**Helen Rizos**

Australia

**Sally Roberts**

United Kingdom

**Craig Robson**

United Kingdom

**Sonia Rocha**

United Kingdom

**Socorro Maria Rodriguez Pinilla**

Spain

**Sébastien Roger**

France

**Massimo Roncalli**

Italy

**Philippe Ronde**

France

**Zhili Rong**

United States of America

**Ilse Rooman**

Australia

**Daniel Rosenberg**

United States of America

**Robert Roskoski**

United States of America

**Martin Rottenberg**

Sweden

**William Roush**

United States of America

**Carlos Rovira**

Sweden

**Jeffrey Rubin**

United States of America

**Mark Russell**

United Kingdom

**Antonio Russo**

Italy

**Fred Sablitzky**

United Kingdom

**Anguraj Sadanandam**

United Kingdom

**Cyrus Safinya**

United States of America

**Susmita Sahoo**

United States of America

**Neveen Said**

United States of America

**Akira Saito**

Japan

**Hiroaki Sakurai**

Japan

**María Salazar**

Spain

**Andrea Salmaggi**

Italy

**Ashok Saluja**

United States of America

**Rajeev Samant**

United States of America

**Deepa Sampath**

United States of America

**Oltea Sampetrean**

Japan

**David Santamaria**

Spain

**Giorgio Santoni**

Italy

**Massimo Santoro**

Italy

**Takashi Sasayama**

Japan

**Fuminori Sati**

Japan

**Shoko Sato**

Japan

**Yuichiro Sato**

Japan

**Nicholas Saunders**

Australia

**Stefania Scala**

Italy

**Inga-Marie Schaefer**

Germany

**Liliana Schaefer**

Germany

**Zachary Schafer**

United States of America

**Daniela Schilling**

Germany

**Johannes Schmid**

Austria

**Laura Schmidt**

United States of America

**Guenter Schneider**

Germany

**Sallie Schneider**

United States of America

**Johannes Schulte**

Germany

**Gregg Semenza**

United States of America

**Shantibushan Senapati**

India

**Peter Senter**

United States of America

**Parthasarathy Seshacharyulu**

United States of America

**Claudio Sette**

Italy

**Mark Shackleton**

Australia

**Kenneth Shain**

United States of America

**Ayesha Shajahan-Haq**

United States of America

**Rajesh Sharan**

India

**Nima Sharifi**

United States of America

**Dipali Sharma**

United States of America

**Hongbing Shen**

China

**Lalita Shevde-Samant**

United States of America

**Hubing Shi**

United States of America

**Jian Shi**

United States of America

**Lei Shi**

China

**Masabumi Shibuya**

Japan

**Vivian Shin**

Hong Kong

**Takashi Shingu**

United States of America

**Atsushi Shiozaki**

Japan

**Adam Shlien**

Canada

**Robert Shoemaker**

United States of America

**Viji Shridhar**

United States of America

**Yongqian Shu**

China

**Arti Shukla**

United States of America

**Markus Siegelin**

United States of America

**Michele Signore**

Italy

**Robert Silverman**

United States of America

**Cherie Singer**

United States of America

**Amar Singh**

United States of America

**Ajay Singh**

United States of America

**Dharmendra Singh**

United States of America

**Pankaj Singh**

United States of America

**Rakesh Singh**

United States of America

**Seema Singh**

United States of America

**Bhawna sirohi**

India

**Nikolajs Sjakste**

Latvia

**Jill Slack-Davis**

United States of America

**Ana Slipicevic**

Norway

**DeeDee Smart**

United States of America

**Degang Song**

United States of America

**Jianguo Song**

China

**Guru Sonpavde**

United States of America

**Richie Soong**

Singapore

**Rhonda Souza**

United States of America

**Giulio C. Spagnoli**

Switzerland

**Riccardo Spizzo**

Italy

**Sanjay K. Srivastava**

United States of America

**Peter Stacpoole**

United States of America

**Olle Stal**

Sweden

**Daniel Starczynowski**

United States of America

**Sandra Steffens**

Germany

**Ulrike Stein**

Germany

**Carsten Stephan**

Germany

**Ursula Stochaj**

Canada

**Christian Stock**

Germany

**Deborah Stroka**

Switzerland

**Thorsten Stühmer**

Germany

**Dwayne Stupack**

United States of America

**Saraswati Sukumar**

United States of America

**Yan Sun**

China

**Luyang sun**

China

**Robyn Sutherland**

Australia

**Hiromu Suzuki**

Japan

**Yoshihiro Suzuki-Karasaki**

Japan

**Mikael Svensson**

Sweden

**Elda Tagliabue**

Italy

**Chiaki Takahashi**

Japan

**Fuyuhiko Tamanoi**

United States of America

**Poh Seng Tan**

Singapore

**Shinji Tanaka**

Japan

**Reshma Taneja**

Singapore

**Faqing Tang**

China

**Takanobu Taniguchi**

Japan

**Hou-Quan Tao**

China

**Maria Angels Tapia-Laliena**

Germany

**Elisa Tassano**

Italy

**Ada Maria Tata**

Italy

**Hussein Tawbi**

United States of America

**Cormac Taylor**

Ireland

**Kathryn Taylor**

United Kingdom

**Luba Tchertanov**

France

**Peter ten Dijke**

Netherlands

**Vinay Tergaonkar**

Singapore

**Luigi Terracciano**

Switzerland

**Stephane Terry**

France

**George Thalmann**

Switzerland

**Christoforos Thomas**

United States of America

**Chenxi Tian**

United States of America

**Randal Tibbetts**

United States of America

**Gregory Tietjen**

United States of America

**Ingeborg Tinhofer**

Germany

**Verena Maria Tischler**

Switzerland

**Dean Tolan**

United States of America

**Ivan Topisirovic**

Canada

**Nobuyuki Toshikuni**

Japan

**Sophie Toya**

United States of America

**Marianna Trakala**

Spain

**Mark Trifiro**

Canada

**Tudorita Tumbar**

United States of America

**Musaffe Tuna**

United States of America

**Masuko Ushio-Fukai**

United States of America

**Katsuhiro Uzawa**

Japan

**Remco van Doorn**

Netherlands

**Matthew Vander Heiden**

United States of America

**Petr Vanhara**

Czech Republic

**Franz Varga**

Austria

**Sudhir Varma**

United States of America

**Edo Vellenga**

Netherlands

**Pasquale Verde**

Italy

**Gyorgy Vereb**

Hungary

**Lars Vereecke**

Belgium

**Suresh Kumar Verma**

United States of America

**Alejandro Villagra**

United States of America

**Carlo Visco**

Italy

**Rebecca Voltan**

Italy

**Peter Vrana**

United States of America

**Paul Wade**

United States of America

**Pat Wadsworth**

United States of America

**Bridget Wagner**

United States of America

**Matthew Wakefield**

Australia

**Hans Wandall**

Denmark

**Harold Wanebo**

United States of America

**Chun-Chao Wang**

United States of America

**Guoxing Wang**

United States of America

**Huamin Wang**

United States of America

**Hongyang Wang**

China

**Jing Wang**

United States of America

**Ji Ming Wang**

United States of America

**Yucai Wang**

United States of America

**Xiaowei Wang**

United States of America

**Yan Wang**

China

**Kishore Wary**

United States of America

**Toshiki Watanabe**

Japan

**Stephen Waxman**

United States of America

**Daniel Weber**

Germany

**Jun Wei**

United States of America

**Britta Weigelt**

United Kingdom

**Michael Weil**

United States of America

**Andrew Weinberg**

United States of America

**Jonathan Weitzman**

France

**Michael Wendt**

United States of America

**Meir Wetzler**

United States of America

**Erik Wiemer**

Netherlands

**David Williams**

United States of America

**Jason Willis**

United States of America

**Brice Wilson**

United States of America

**Ido Wolf**

Israel

**Georg Wondrak**

United States of America

**Kwong-Kwok Wong**

United States of America

**Mark Wong**

Australia

**Gen Wu**

United States of America

**Xiangwei Wu**

United States of America

**Yadi Wu**

United States of America

**Guo Xiaobo**

China

**Ke Xu**

China

**Liang Xu**

United States of America

**Qilin Xu**

United States of America

**Nagendra Yadava**

United States of America

**Nobuo Yaegashi**

Japan

**Ryuji Yamaguchi**

Japan

**Munekazu Yamakuchi**

Japan

**Soichiro Yamamura**

United States of America

**Min Yan**

United States of America

**Burton Yang**

Canada

**Jin-Ming Yang**

United States of America

**Ruey-Bing Yang**

Taiwan

**Yong Yang**

China

**Yanan Yang**

United States of America

**Hua Yang**

China

**Ming Yang**

China

**Young Yang**

Korea South

**Zeng-jie Yang**

United States of America

**Jason Yap**

United Kingdom

**Chueh-Chuan Yen**

Taiwan

**Venkat Laxmi Yeruva**

United States of America

**Junji Yodoi**

Japan

**Takehiko Yokobori**

Japan

**Takashi Yokota**

Japan

**Yukio Yoneda**

Japan

**Young Yoo**

Korea South

**Go Yoshida**

Japan

**Kosuke Yoshihara**

Japan

**Liang You**

United States of America

**Lawrence Young**

United Kingdom

**Shi-cang Yu**

China

**Xiaozhong Yu**

United States of America

**Zhang Yu**

China

**Zhong Yun**

United States of America

**Peter Zage**

United States of America

**Stefan Zahler**

Germany

**Roza Zakany**

Hungary

**Gerard Zambetti**

United States of America

**Hamid Zand**

Iran

**Mengwei Zang**

United States of America

**Giorgio Zauli**

Italy

**Jean-Claude Zenklusen**

United States of America

**Ann Zeuner**

Italy

**Jose Zevallos**

United States of America

**Dong-Er Zhang**

United States of America

**Jian-Ting Zhang**

United States of America

**Wei Zhang**

Singapore

**Wei Zhang**

United States of America

**Xu Dong Zhang**

Australia

**Xiaohong Zhang**

United States of America

**Lingqiang Zhang**

China

**Lin Zhang**

United States of America

**Wei Zhang**

China

**Yongsheng Zhang**

China

**Jianhua Zhang**

United States of America

**Zhiyong Zhang**

United States of America

**Chen Zhao**

United States of America

**Lingyu Zhao**

China

**Zhihe Zhao**

China

**Jun-Nian Zheng**

China

**Suting Zheng**

United States of America

**Wenxin Zheng**

United States of America

**Minghao Zhong**

United States of America

**Weide Zhong**

China

**Xiaomin Zhong**

China

**Chunyan Zhou**

China

**Jianwei Zhou**

China

**Binhua Zhou**

United States of America

**Yu-Dong Zhou**

United Kingdom

**Yi-Hong Zhou**

United States of America

**Yunli Zhou**

United States of America

**Qinbo Zhou**

United States of America

**ChangQi Zhu**

Canada

**Jay-Jiguang Zhu**

United States of America

**Wei-Guo Zhu**

China

**Zhengping Zhuang**

United States of America

**Inti Zlobec**

Switzerland

**Massimo Zollo**

Italy

**Wei-Xing Zong**

United States of America

